# Migraine evolution after the cessation of CGRP(-receptor) antibody prophylaxis: a prospective, longitudinal cohort study

**DOI:** 10.1177/03331024211046617

**Published:** 2021-09-27

**Authors:** Bianca Raffaelli, Maria Terhart, Lucas Hendrik Overeem, Jasper Mecklenburg, Lars Neeb, Maureen Steinicke, Uwe Reuter

**Affiliations:** 1 Department of Neurology, Charité - Universitätsmedizin Berlin, Berlin, Germany; 2 Universitätsmedizin Greifswald, Greifswald, Germany

**Keywords:** Migraine, CGRP, monoclonal antibodies, discontinuation

## Abstract

**Background:**

National and international guidelines recommend stopping migraine prophylaxis with CGRP(-receptor) monoclonal antibodies after 6–12 months of successful therapy. In this study, we aimed to analyze the course of migraine for four months after the cessation of CGRP(-receptor) antibodies use.

**Methods:**

This longitudinal cohort study included patients with migraine who received a CGRP-(receptor) antibody for ≥8 months before treatment cessation. We analyzed headache data in the four-week period prior to mAb treatment initiation (baseline), in the month before the last mAb injection, in weeks 5–8 and 13–16 after last treatment. Primary endpoint of the study was the change of monthly migraine days from the month before last treatment to weeks 13–16. Secondary endpoints were changes in monthly headache days and monthly days with acute medication use.

**Results:**

A total of 62 patients equally distributed between prophylaxis with the CGRP-receptor antibody erenumab and the CGRP antibodies galcanezumab or fremanezumab participated in the study. Patients reported 8.2 ± 6.6 monthly migraine days in the month before last treatment. Monthly migraine days gradually increased to 10.3 ± 6.8 in weeks 5–8 (p = 0.001) and to 12.5 ± 6.6 in weeks 13–16 (p < 0.001) after drug cessation. Monthly migraine days in weeks 13–16 were not different from baseline values (−0.8 ± 5.4; p > 0.999). Monthly headache days and monthly days with acute medication use showed a similar pattern.

**Conclusions:**

The cessation of CGRP(-receptor) antibodies migraine prophylaxis was associated with a significant increase of migraine frequency and acute medication intake over time.

## Introduction

Monoclonal antibodies (mAbs) targeting Calcitonin Gene-Related Peptide (CGRP) and its receptor are the first drugs specifically designed for the prophylactic treatment of migraine (1). They have fundamentally improved our therapeutic armamentarium against this sometimes severe and disabling headache disorder with good efficacy even in patients with several prior non-successful treatment attempts (1–6). Their safety and tolerability profile is excellent and superior to those of other oral preventatives (7). However, the ideal treatment duration with these two CGRP(-receptor) mAb classes in real life has yet to be determined. It is also unclear if a prolonged benefit on migraine can be expected after treatment discontinuation, which would point to a disease-modifying character of these substances.

The European Headache Federation (EHF) treatment guidelines and several national societies suggest stopping prophylactic therapy with a CGRP(-receptor) mAb after 6–12 months of successful treatment (8). This suggestion is based on expert opinion, in line with the recommendations for oral migraine prophylactic medications.

In the majority of clinical trials, patients were treated with mAbs for 6–12 months (9–14) with limited data on the evolution of migraine after treatment termination. A follow-up analysis in patients with episodic migraine (EM) from two randomized trials with galcanezumab (EVOLVE 1 + 2) and a duration of six months revealed a marginal worsening of the disease after study completion (15). Migraine frequency remained significantly lower than before randomization for up to four months after the last drug injection (15). A small series of patients with chronic migraine (CM) receiving the CGRP-receptor mAb erenumab or galcanezumab in the open label extension phase in two clinical trials showed sustained efficacy for three months after trial completion, although with a small increase of monthly migraine days over time (16). Real-world evidence on the course of migraine after mAb treatment termination is scarce and limited to erenumab (17,18).

In this study, we aimed to assess the course of the disease after cessation of migraine prophylaxis with the CGRP-receptor mAb erenumab and the CGRP mAbs galcanezumab and fremanezumab.

## Methods

### Study design and participants

This was a longitudinal cohort study conducted at the Headache Center, Charité – Universitätsmedizin Berlin. We included adult patients with migraine on prophylactic therapy with a CGRP(-receptor) mAb. All patients were scheduled to discontinue prophylaxis in line with the European Headache Federation and German treatment guidelines. Migraine was diagnosed according to the ICHD-3 classification based on the year prior to mAb treatment initiation (19). The cohort in this manuscript consisted of migraine patients who had been treated unsuccessfully (i.e. insufficient efficacy or poor tolerability) or had contraindications for all first-line oral preventive treatments (beta blockers, topiramate, flunarizine, and amitriptyline) as listed by the German authorities, and additionally onabotulinumtoxinA in CM.

For study inclusion, patients had to have a minimum of 8 mAb injections received with a frequency of one injection per month. Patients in this study had to be on the first prophylactic treatment with a CGRP mAb and on monotherapy. A further inclusion criterion was a sustained benefit from mAb treatment as determined by the patient.

We divided patients into two groups: 1) receptor group: patients who received the CGRP-receptor mAb erenumab (70 mg or 140 mg subcutaneous monthly) and 2) ligand group: patients who received one of the CGRP mAbs: galcanezumab (240 mg loading dose and then 120 mg sc monthly) or fremanezumab (225 mg sc monthly). The substance (mAb) choice was made at the discretion of the treating neurologist.

### Study procedures

**Figure 1:**
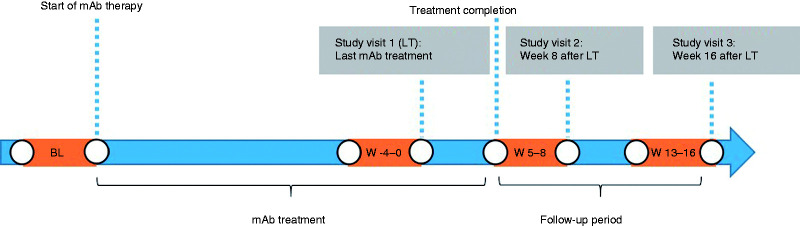
Study timeline. The periods marked in orange correspond to the weeks that were analyzed for the study. BL = baseline, LT = last treatment.

All patients who were scheduled for medication pause were contacted by telephone four weeks prior to the last mAb injection (= last treatment, LT), and reminded to carefully record their headache data on a daily basis for the following weeks. Patients were also scheduled for their first study visit. The study consisted of three prospective study visits: In visit one, patients received the last mAb injection (LT). The second visit was planned after eight weeks and the third visits 16 weeks after LT (Figure 1).

Baseline headache data for the four-week period prior to the start of the mAb therapy was collected retrospectively from the patients’ electronic chart. Complete headache data is a prerequisite for the first mAb administration in our Headache Center.

At visit 1 (= LT), we checked inclusion and exclusion criteria, obtained informed consent and collected the following demographic and anamnestic data: age, sex, years since first migraine manifestation, occurrence of migraine aura (yes/no), and number of months under mAb treatment. We then recorded the prospective headache information of the previous four weeks from standardized headache diaries. Patients are routinely instructed to bring their current headache diary to every appointment. The headache information included the number of monthly migraine days (MMD), monthly headache days (MHD), and monthly days with the use of acute medication (AMD).

A migraine day was defined as any calendar day with a headache fulfilling the criteria of a definite or probable migraine according to the ICHD-3 classification (19). Both triptans and non-triptan pain medication, e.g. nonsteroidal anti-inflammatory drug (NSAID), counted as acute medication. In accordance with randomized controlled trials for CGRP(-receptor) mAbs (11,20), a headache day with the intake of a triptan was also classified as a migraine day, regardless of the headache duration, intensity or accompanying symptoms.

Migraine frequency was stratified in very low frequency episodic migraine (VLFEM = < 4 migraine days/month), low frequency episodic migraine (LFEM = 4–7 migraine days/month), high frequency episodic migraine (HFEM = ≥8 migraine days/month and <15 headache days/month), and chronic migraine (CM = ≥8 migraine days/month and ≥15 headache days/month).

Headache parameters were also collected for weeks 5–8 and weeks 13–16 after LT. If a patient restarted mAb treatment before week 16, data of visit two were transferred to visit three, following the last-observation-carried-forward methodology.

### Outcomes and endpoints

Primary outcomes of the study were the number of MMD at baseline and at every study visit in all patients and both groups separately. Secondary outcomes were the number of MHD, AMD and the 30% and 50% responder rates, defined as the proportion of patients with an improvement in MMD of ≥30% or ≥50% from baseline.

Primary endpoint was the change of MMD from LT (weeks -4-0) to visit three (weeks 13-16) in the entire study population, and in both study groups separately. Secondary endpoints were the changes in MMD from LT to visit two (weeks 5–8) and from baseline to all time points as well as the changes in MHD, AMD and responder rates over time.

### Statistical analysis

Sample size calculation was performed using the software nQuery Advisor 7.0. We expected a similar worsening of MMD three months after treatment discontinuation in both the receptor and the ligand group with a maximum difference of 2.5 MMD between groups (irrelevance margin). Based on a previous study, we further assume a standard deviation of ±3.10 in each group (21). With a sample size of 28 patients per group, the difference of MMD between groups is completely contained in the assumed interval [−2.5; 2.5] with a statistical power of 80% at a significance level of α = 0.05 (two-tailed). Assuming a drop-out rate of 10%, we therefore planned to enroll 31 patients per group.

Demographic and anamnestic data was summarized using descriptive statistics (frequencies and percentages for categorical variables, mean ± standard deviation for numerical variables). We tested the primary and secondary outcome variables for normal distribution using the Kolgomorov-Smirnov test. Since the data was not normally distributed, we compared outcomes using non-parametric tests, i.e. Friedman test with post hoc pairwise comparisons for dependent samples or Mann-Whitney U test for independent samples. A two-tailed p value <0.05 was considered statistically significant. P values were adjusted for multiple comparisons using the Bonferroni method.

All statistical analyses were performed using SPSS 25 (IBM, NY, USA).

#### Ethics

The Charité Ethical Committee (EA1/274/19) approved the study. All participants gave written informed consent following study information.

## Results

### Demographics and patients’ characteristics

We enrolled n = 62 participants between January and November 2020 in the study with an equal distribution of patients on erenumab (n = 31) and patients on galcanezumab or fremanezumab (n = 31) for migraine prophylaxis. A total of n = 29 patients in the receptor mAb group (93.5%) and n = 30 patients (96.8%) in the CGRP mAb group completed the study, matching our sample size calculation. In the CGRP-receptor group, two patients were lost to follow-up. In the ligand group, one patient withdrew consent to participate.

Baseline characteristics are illustrated in Table 1. During the four-week period prior to the start of the mAb therapy (baseline), patients reported 13.3 ± 6.4 MMD, 15.1 ± 7.0 MHD and 10.4 ± 5.6 AMD. Most patients (62.7%) fulfilled the criteria of CM in the year prior to treatment initiation (19).

**Table 1:** Demographics characteristics of study participants. Values are mean ± standards deviation or n (%).

	CGRP-receptor mAb-group	CGRP mAb-group	p value
n	29 • n = 9: 70 mg• n = 20: 140 mg	30• n = 20: galcanezumab• n = 10: fremanezumab	
Age (years)	49.3 ± 12.9	49.2 ± 10.1	0.99
Sex (female)	25 (89.7%)	29 (96.7%)	0.35
Chronic migraine	20 (69.0%)	22 (73.3%)	0.78
With aura	17 (58.6%)	18 (60.0%)	>0.999
Years since first manifestation of migraine	28.7 ± 11.3	30.8 ± 11.8	0.50
Months of treatment before discontinuation	9.8 ± 1.3	9.5 ± 0.8	0.41

Prior to treatment discontinuation, patients received CGRP(-receptor) mAb prophylaxis for 9.7 ± 1.1 treatment cycles with drug administration every four weeks. Both groups were similar in age, sex, migraine type and treatment duration (Table 1).

### Migraine evolution after treatment cessation

In the four-week period before LT, patients recorded 8.2 ± 6.6 MMD.

The MMD increased to 10.3 ± 6.8 in weeks 5–8 (p = 0.001 vs. LT) and to 12.5 ± 6.6 in weeks 13–16 (p < 0.001 vs. LT). MHD and AMD showed a similar pattern with a gradual deterioration beginning in weeks 5-8 (Table 2).

**Table 2:** Monthly migraine days, monthly headache days, monthly days with acute medication use, and migraine frequency subgroups in the four weeks before treatment begin (baseline), before the last mAb injection (LT period) and after treatment discontinuation for all patients. Values are mean ± standard deviation. p values are provided for the primary and secondary endpoints.

	Baseline	LT period (weeks -4-0)	Weeks 5–8	Weeks 13–16
Monthly migraine days	13.3 ± 6.4	8.2 ± 6.6	10.3 ± 6.8	12.5 ± 6.6
vs. LT period			p = 0.001*	p < 0.001*
vs. baseline		p < 0.001*	p = 0.033*	p > 0.999
Monthly headache days	15.1 ± 7.0	9.4 ± 7.3	11.4 ± 7.3	13.4 ± 6.7
vs. LT period			p = 0.001*	p < 0.001*
vs. baseline		p = 0.001*	p = 0.010*	p > 0.999
Monthly days with acute medication use	10.4 ± 5.6	5.9 ± 5.0	7.7 ± 6.2	9.3 ± 6.3
p vs. LT period			p = 0.008*	p < 0.001*
p vs. baseline		p < 0.001*	p = 0.001*	p = 0.32
Migraine frequency subgroups				
VLFEM (n, %)	0 (0%)	15 (25.4%)	6 (10.2%)	2 (3.4%)
LFEM (n, %)	7 (11.8%)	18 (30.5%)	20 (33.9%)	14 (23.7%)
HFEM (n, %)	28 (47.5%)	16 (27.1%)	17 (28.8%)	18 (30.5%)
CM (n, %)	24 (40.7%)	10 (17.0%)	16 (27.1%)	25 (43.4%)

* = statistically significant. VLFEM = very low frequency episodic migraine (<4 migraine days/month); LFEM = low frequency episodic migraine (4–7 migraine days/month); HFEM = high frequency episodic migraine/chronic migraine (≥8 migraine days/month and <15 headache days/month); CM = chronic migraine (≥8 migraine days/month and ≥15 headache days/month).

Compared to patients with CM, patients with LFEM had a higher increase in MMD (p = 0.024) and MHD (p = 0.010) in weeks 13–16 after treatment cessation (Supplemental Table S1).

Subgroup analyses revealed a more rapid deterioration in patients with erenumab than in patients with a CGRP mAb (Supplemental Table S2): In the erenumab group, MMDs increased from 8.7 ± 5.9 to 11.5 ± 6.2 in weeks 5–8 (p = 0.001 vs. LT), while there was no statistical difference between LT and weeks 5–8 in the CGRP mAb group (7.8 ± 7.3 vs. 9.1 ± 7.3, p > 0.999). During weeks 13–16, both groups reported a significant increase in MMD compared to LT with 13.3 ± 6.0 MMDs in the erenumab group (p < 0.001 vs. LT) and 11.7 ± 7.2 MMDs in the CGRP mAb group (p = 0.003 vs. LT) with no differences between groups (p > 0.999).

Eight patients (13.8%, n = 6 in the receptor group and n = 2 in the ligand group) started mAb treatment again before the third study visit, while the others remained without preventive treatment until week 16. However, the great majority of patients (n = 54, 91.5%) restarted mAb therapy at the end of this study.

### Migraine evolution in comparison to baseline

Compared to baseline, MMDs decreased by −5.0 ± 6.0 in the LT period (p < 0.001). Migraine frequency remained significantly lower than during baseline in weeks 5–8 (−2.9 ± 6.4 MMD, p = 0.033), but returned to baseline levels during weeks 13–16 (−0.8 ± 5.4 MMD, p > 0.999).

Patients in both groups benefited similarly from mAb therapy: patients on erenumab reported a decrease by −4.9 ± 5.5 in the four-week period before LT (p < 0.001), while patients with a CGRP mAb improved by −5.1 ± 6.6 (p < 0.001).

**Figure 2:**
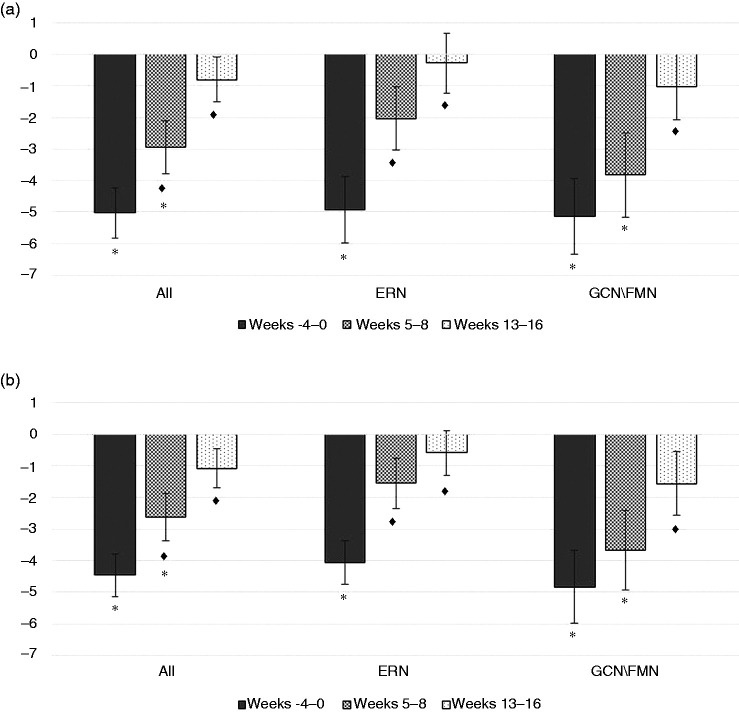
Absolute changes of monthly migraine days (a), and monthly days with acute medication use (b) compared to baseline during the last mAb treatment month (weeks -4–0) and after treatment discontinuation for all patients, patients with erenumab and patients with galcanezumab/fremanezumab. Values are illustrated as mean ± standard error. ERN = erenumab. GCN/FMN = galcanezumab/fremanezumab. ♦ = significant vs. weeks -4-0. * = significant vs. baseline.

Already two months after cessation, migraine frequency was similar to baseline values in the erenumab group (p > 0.999). In contrast, patients in the CGRP mAb group reached baseline levels later in weeks 13–16 (p > 0.999; Figure 2).

### Responder rates

In the four week period before LT, two-thirds of patients (n = 38, 64.4%) had a reduction of MMD of ≥30% and almost half of patients (n = 29, 49.1%) of ≥50% compared to baseline. Eight patients (13.6%) had a reduction of MMD between 10 and 30%, while 22.0% (n = 13) reported a subjective improvement, which was not reflected by a reduction of MMD.

Responder rates decreased significantly after treatment cessation. During weeks 13–16, only 22.0% of patients had a reduction ≥30% compared to baseline and only 10.2% a reduction ≥50%.

Subgroup analysis of the ≥30% and <30% responders in LT revealed a significant worsening of MMD, MHD and AMD after discontinuation in both groups (p < 0.05 vs. LT for both groups). A numerical greater increase of all parameters was seen in the ≥30% responders (p > 0.05, not significant) (Supplemental Table S3).

## Discussion

The cessation of migraine prophylaxis with CGRP-(receptor) monoclonal antibodies was associated with a continuous increase of migraine frequency over time. After four months, the majority of patients were back to baseline migraine frequency prior to the start of prophylaxis. Monthly headache days and monthly days with acute medication use increased in parallel over time. Patients previously on erenumab showed a faster deterioration than patients with previous galcanezumab or fremanezumab therapy. The 30% responder rate, which was 64% in the last four weeks of active treatment, decreased rapidly to 22% four months after the last mAb injection.

This real-word data shows a more pronounced deterioration of migraine after treatment cessation than data after the termination of the galcanezumab trials (EVOLVE 1 and 2) in EM patients (15). Three months after termination of the double-blind study phase, patients reported only one MMD more than during the last treatment month and remained significantly below baseline levels (15). An analysis of 16 patients with CM, who completed the open-label phases of clinical trials with erenumab and galcanezumab showed a numerical increase by approximately two MMD in the third month after trial completion without a significant difference to the last trial month (16). In contrast, this larger study shows an increase of more than four MMD in the same observation period and a return to baseline values. Different settings (clinical trial vs. real world) may contribute to the differences: in particular, patients in our study had the possibility to restart mAb treatment, if needed, which was not possible in the follow-up period of the randomized trials. Also, most of our patients had significantly more MMD before treatment initiation than in the EVOLVE studies (14,22). This cohort had failed all preventive treatments of first choice in Germany, whereas failure of several drug classes led to exclusion from most clinical trials, including both EVOLVE studies (14,22).

The four-week real-life observation by De Matteis et al. described a significant increase in MMD and AMD already in the first month after stopping erenumab (17). In a recent retrospective study from Switzerland by Gantenbein et al., 25 of 28 patients showed an increase of MMD in the third month after erenumab discontinuation (18). Our data confirm these results. The difference of this study relates to a prospective approach and the inclusion of patients on galcanezumab and fremanezumab. The analysis of responder rates and the stratification of migraine frequency represents another new aspect of this research.

This analysis revealed a different time course after treatment cessation between patients previously treated with erenumab and patients treated with CGRP mAbs. The faster aggravation after stopping erenumab could be related to its shorter elimination half-life time, which is about 21 days (23). Galcanezumab and fremanezumab have a longer bioavailability with half-life times of 27 and 30 days, respectively (24,25). However, this difference was only temporary and both groups were back to baseline migraine frequency four months after the termination of prophylaxis.

The aim of prophylaxis is the reduction of migraine frequency and pain intensity resulting in improved quality of life. On a pathophysiological level, the goal is to normalize the underlying neuronal dysfunction, which would result in a long-lasting disease modification and persistent effects after treatment cessation. Some studies described ongoing benefits for oral preventives with a mode of action within the central nervous system (CNS) such as flunarizine, metoprolol and propranolol, and an enduring reduction of MMD for 6-8 months after treatment cessation (26,27). One placebo-controlled trial investigated the effects of topiramate discontinuation on migraine frequency (28): after receiving topiramate for six months, 514 patients were randomized to continue with topiramate or placebo for a further six months. During the last trial month, the increase in MMD was greater in the placebo group (+1.19, 95% CI 0.71 to 1.66) than in the topiramate group (+0.10, 95% CI −0.36 to 0.56). Patients in the placebo group maintained a significant improvement in MMD and did not return to baseline levels (pre-open-label), indicating a protracted benefit of topiramate after treatment discontinuation (28). In contrast, most patients in our analysis did not experience ongoing benefits after CGRP(-receptor) mAbs treatment cessation. Only 10% of patients had a sustained treatment response of ≥50% in the fourth months after treatment discontinuation and only 8% decided to continue the drug holiday after week 16.

Changes in neuronal networks under treatment with CGRP-(receptor) mAbs as recently shown in an MRI functional imaging study (29) may not result in long-term improvement of migraine after 8–12 months of therapy. The magnitude of a central response may account for the differences between mAbs and oral preventatives as mAbs do not cross the blood-brain barrier easily (30). The peripheral inhibition of meningeal nociception by CGRP(-receptor) mAbs may only lead to short-lasting network changes (31).

The results of this analysis comprising 60 patients raise the question whether cessation of treatment should be recommended in all patients on migraine prophylaxis with CGRP-(receptor) mAbs. The main reasons for treatment discontinuation with oral preventives were emerging side effects and an unfavorable risk-benefit profile (32). In contrast, tolerability issues play only a minor role with CGRP targeted mAb therapies. In an open-label clinical trial with patients on erenumab prophylaxis for five years, adverse events did not increase over time and remained similar to what was observed in the placebo group during the parent study (33).

While our data indicate that a drug holiday can lead to a disease deterioration, some arguments still exist in favor of a discontinuation attempt. Most importantly, migraine frequency can fluctuate during the course of life (34). The interruption of a prophylactic treatment is useful to detect a natural improvement and to periodically reassess the need for prevention. From an economic perspective, high monthly mAb treatment costs should also be taken into account.

This is the first prospective real-world analysis assessing treatment cessation over four months in a larger number of patients with substances from two different CGRP(-receptor) antibody classes. The main limitation of this study is the uncontrolled setting with the lack of a control group, which is the nature of real-world evidence. The expectation bias could have contributed to the worsening of migraine characteristics after treatment cessation. However, the conditions in this study are intended to provide insights on discontinuation attempts in a real-world setting, where patients are, in fact, subject to bias and nocebo effects.

In conclusion, in the majority of patients, the benefits of migraine prophylaxis with CGRP-(receptor) mAbs were significantly reduced after three months of a drug holiday. Future larger-scale placebo-controlled studies are needed to corroborate our results and also to identify predictors for successful treatment discontinuation.

## Clinical implications

- The discontinuation of migraine prevention with CGRP(-receptor) mAbs was associated with a progressive worsening of migraine over time.
- In most patients, the treatment benefit was significantly reduced four months after the last CGRP(-receptor) mAb injection.
- A discontinuation attempt should be carefully discussed with patients on successful CGRP(-receptor) mAbs therapy on an individual basis.
